# Magnitude of phonetic distinction predicts success at early word learning in native and non-native accents

**DOI:** 10.3389/fpsyg.2014.01059

**Published:** 2014-09-30

**Authors:** Paola Escudero, Catherine T. Best, Christine Kitamura, Karen E. Mulak

**Affiliations:** ^1^The MARCS Institute, University of Western SydneySydney, NSW, Australia; ^2^School of Social Sciences and Psychology, University of Western SydneySydney, NSW, Australia

**Keywords:** early word learning, phonetic distinction, native accent, non-native accent, vowel perception

## Abstract

Although infants perceptually attune to native vowels and consonants well before 12 months, at 13–15 months, they have difficulty learning to associate novel words that differ by their initial consonant (e.g., BIN and DIN) to their visual referents. However, this difficulty may not apply to all minimal pair novel words. While Canadian English (CE) 15-month-olds failed to respond to a switch from the newly learned word DEET to the novel non-word DOOT, they did notice a switch from DEET to DIT (Curtin et al., 2009). Those authors argued that early word learners capitalize on large phonetic differences, seen in CE DEET–DIT, but not on smaller phonetic differences, as in CE DEET–DOOT. To assess this hypothesis, we tested Australian English (AusE) 15-month-olds, as AusE has a smaller magnitude of phonetic difference in both novel word pairs. Two groups of infants were trained on the novel word DEET and tested on the vowel switches in DIT and DOOT, produced by an AusE female speaker or the same CE female speaker as in Curtin et al. (2009). If the size of the phonetic distinction plays a more central role than native accent experience in early word learning, AusE children should more easily recognize both of the unfamiliar but larger CE vowel switches than the more familiar but smaller AusE ones. The results support our phonetic-magnitude hypothesis: AusE children taught and tested with the CE-accented novel words looked longer to both of the switch test trials (DIT, DOOT) than same test trials (DEET), while those who heard the AusE-accented tokens did not notice either switch. Implications of our findings for models of early word learning are discussed.

## INTRODUCTION

The first year of life sees the emergence of native phonemic categories, demonstrated by children’s persisting discrimination of native contrasts and diminishing discrimination of non-native contrasts (Werker and Tees, 1983, 1984; Polka and Werker, 1994). Children are born able to discriminate nearly all consonant and vowel contrasts (e.g., Aslin and Pisoni, 1980; for reviews, see Burnham, 1986; Best, 1994; Werker and Tees, 1999), but by 6–8 months this ability begins to decline for many vowel contrasts not present in the native language environment (Polka and Werker, 1994; cf. Polka and Bohn, 1996), and by 10–12 months sensitivity to most non-native consonant contrasts similarly declines (Werker and Tees, 1983, 1984; cf. Best et al., 1988, 1995). For instance, infants aged 6–8 months brought up in an English language environment discriminate the Hindi contrast [ta]–[a] and Salish contrast [k’i]-[q’i], but by 10–12 months this ability declines, and continues to do so until, like English-speaking adults, they are no longer able to reliably discriminate many contrasts that are not present in their native language environment. By the same token, children brought up in Hindi or Salish language environments continue to discriminate the contrasts present in their native languages, as do Hindi-speaking and Salish-speaking adults (Werker and Tees, 1983, 1984).

Paradoxically, following this auspicious beginning, 14-month-old children have difficulty applying their phonetic and phonological knowledge to learning new words. That is, children younger than 17 months do not reliably discriminate newly learned words that differ by a single native consonant contrast (Stager and Werker, 1997; Werker et al., 2002; Pater et al., 2004), whereas older children succeed (Werker et al., 2002). For example, in a Switch task in which infants were habituated to novel word-object pairings, 14-month-olds failed to notice when the novel word associated with one object was switched to a new word that differed in only one consonant (e.g., BIH switched to DIH). Crucially, this was not due to a general problem with associating visual referents to spoken words, because 14-month-olds did learn word-referent pairs when the words differed in all of their consonants and vowels, such as LIF vs. NEEM. Nor was it due to an inability to discriminate the minimal pair contrasts, as 14-month-olds discriminated the same consonant minimal pair words when they were presented outside a word-learning context in a simple auditory discrimination task (Stager and Werker, 1997).

Researchers have suggested that the difficulty children younger than 17 months have in using phonetic detail for the purpose of word learning is due to the circumstances or demands of the experimental task (e.g., Stager and Werker, 1997; Fennell and Werker, 2003). Word learning is argued to be a difficult task, with increased difficulty for similar sounding words (Werker and Fennell, 2004). Indeed, success at associating novel words to visual referents depends on a variety of perceptual, attentional and memory factors (Thiessen, 2007; Rost and McMurray, 2009; Yoshida et al., 2009). For instance, although the 14-month-olds described above failed to notice when a newly learned word was switched to a word differing in one consonant in the Switch task (Stager and Werker, 1997), children’s successful pairing of the novel words BIN and DIN with their corresponding novel objects was demonstrated when they instead performed a preferential looking task after exposure to the associations (Yoshida et al., 2009). Children’s success in learning the novel words BIN and DIN in a preferential looking task but not in a Switch task suggests that the latter is a more demanding task than the former. That is, while children may be able to encode some phonetic detail in novel words, they are unable to do so to an extent that allows them to overcome the additional demands of the Switch task (Yoshida et al., 2009).

Furthermore, contextualization of novel words aids early word learning. Young children learn novel word-object mappings with words that differ in only one consonant when it is clear that the words and objects are to be associated. That is, when presented with sentences such as “Look. It’s the BIN,” or “I like the BIN,” 14-month-olds learn that “BIN” and “DIN” refer to two different objects (Fennell and Waxman, 2010). Accessing phonetic detail in early word learning is also aided by prior exposure to familiar words that refer to familiar objects such as “car” and “kitty,” and prior exposure to the visual referents aids the association of those objects to similar sounding novel words (Fennell, 2012).

Another line of research has shown that not all novel minimal pair words are equally difficult for young children, and that difficulties with some pairs persist beyond the first 2 years of life. In an interactive object-reaching task where children learn to pair novel objects with their novel names, 16-, 20- and 30-month-olds learned and identified novel minimal pairs that differed in only one consonant, but intriguingly, failed with pairs that differed in only one vowel (Nazzi, 2005; Nazzi and New, 2007; Havy and Nazzi, 2009; Nazzi et al., 2009). This consonant-vowel disparity is found even when the cognitive demand is reduced by testing children on familiar words. In a preferential-looking task, 15-month-olds were sensitive to consonant mispronunciations of familiar words (e.g., BALL pronounced GALL), but were less sensitive to vowel mispronunciations (e.g., BALL pronounced BULE; Mani and Plunkett, 2007). In the same experiment, 18-month-olds (and 24-month-olds) were sensitive to both consonant and vowel mispronunciations of familiar words, converging with research demonstrating sensitivity at that age to lexically contrastive variation in vowels embedded in novel words (Dietrich et al., 2007).

Tasks that are more supportive and provide more context about words and their referents have been shown to decrease cognitive task demands, resulting in successful novel word learning by children younger than 17 months (Fennell and Waxman, 2010). The interactive object-reaching task (Nazzi, 2005; Nazzi and New, 2007; Havy and Nazzi, 2009; Nazzi et al., 2009), which presents words in a sentential context and allows pre-exposure to items before each trial, is thus reasoned to impose lower cognitive demands relative to the Switch task. Havy and Nazzi’s (2009) finding that 16-month-olds were able to learn novel minimal pairs differing in only one consonant in an interactive object-reaching task further supports the notion that similarly aged infants’ failure to learn novel minimal pair words in the Switch task is due to its higher cognitive demands, which lead to an underrepresentation of infants’ abilities (Yoshida et al., 2009). But even when tested in procedures thought to impose relatively lower cognitive demands, such as the interactive object-reaching task and the preferential looking tasks used by Mani and Plunkett (2007), children younger than 18 months do not reliably learn novel word-object associations involving vowel minimal pairs. This suggests that a greater difficulty with vowel minimal pairs relative to consonant minimal pairs for children younger than 17 months would persist if tested in the Switch task. Also, the fact that no single vowel minimal pair was correctly identified by the 16-month-olds in Havy and Nazzi (2009) suggests that this difficulty might extend to all vowel minimal pairs. These predictions are in line with Nespor et al.’s (2003) hypothesis that infants should focus more on consonants than vowels in early word learning because vowels carry more between-speaker variation and are perceived less categorically (e.g., Pisoni, 1973).

However, infants younger than 17 months *have* learned some novel vowel minimal pairs in a Switch paradigm. Curtin et al. (2009) found that Canadian English (CE) learning 15-month-olds associated two novel words that differed in only one CE vowel to their corresponding novel object referents in the Switch task. Using the same version of the Switch task as that used by Werker et al. (2002), three groups of children were trained on two novel word-object associations for one of three vowel minimal pairs: DEET–DIT, DEET–DOOT, and DIT–DOOT. At test, only the group presented with DEET–DIT noticed a switch in the word-object pairing (Switch trials), as shown by their higher looking time relative to trials that presented the prior word-object associations (Same trials). Children in the DEET–DOOT and DIT–DOOT training conditions did not demonstrate a difference in looking time to Switch trials vs. Same trials in the test phase, suggesting that only some vowel minimal pairs can be learned under the high demands of the original Switch task.

Curtin et al. (2009) suggested these findings indicate that infants’ phonological representations of vowels may not be adult-like and may instead be based on the most reliable phonetic dimensions for the specific contrast. Vowels are defined by their formant frequencies, which largely reflect the position of the tongue body when producing them. The first formant (F1) is primarily associated with vowel height (tongue height), and in CE, F1 was found to reliably distinguish /i/–// (DEET–DIT) but not the other two non-discriminated vowel contrasts, which were instead reliably differentiated only by F2 (vowel/tongue backness: /i/–/u/ [DEET–DOOT] and //–/u/ [DIT–DOOT]), and F3 (lip rounding: /i/–/u/). That 15-month-olds discriminated only the contrast /i/–// suggests that for young children, the F1 dimension (vowel/tongue height) may be a stronger phonetic cue for distinguishing vowels than F2 and F3. That is, they may take the simpler approach of attending to F1 over attending to a wider range of cues. The authors proposed several reasons for this bias toward F1, which may be more apparent in tasks with high demands. Firstly, F1 may draw more attention simply because it has the most energy in the speech signal. Alternatively, it may be that in the linguistic environment of CE, F1 is attended to most because of the wide range of vowel contrasts that are defined by F1 differences, and furthermore by the weakening of cues such as F2 and F3 due to increased fronting and decreased rounding of the cardinal vowel /u/ in North American English accents (Thomas, 2001; Curtin et al., 2009, p. 5). As the authors pointed out, these interpretations are consistent with the linguistic perception (LP) model (Boersma et al., 2003; Escudero and Boersma, 2004; Escudero, 2005, 2009), which proposes that young children categorize segments according to large and consistent phonetic differences along individual continua, rather than multidimensional phonemic categories as seen in adults, and that only later in development do abstract phonological categories emerge. The findings are also compatible within the framework for processing rich information from multidimensional interactive representations (PRIMIR; Werker and Curtin, 2005), which posits that the reliance on individual phonetic dimensions decreases over time as phonemes emerge.

Curtin et al.’s (2009) findings demonstrate that the magnitude of the phonetic distinction between two vowel sounds is predictive of early word learning success. In the present study, we further examine the phonetic-magnitude hypothesis across two different English accents. We reasoned that children from an English regional accent background [Australian English (AusE)] that displays much smaller phonetic differences among the same three vowels than those presented in CE, and who are unfamiliar with CE, may use the same phonetic dimensions differently. The results of our study will demonstrate whether the F1 dimension is always the phonetic cue that receives most attention regardless of accent differences, or whether the magnitude of its importance is accent-dependent. The results will also shed light on whether success in early word learning is restricted to children’s native accent. We examined AusE 15-month-olds’ ability to learn and discriminate the novel words DEET, DIT and DOOT, comparing performance between participants presented with the words produced in their native AusE accent, and participants presented with words produced in the unfamiliar CE accent. We used the simple version of the Switch task (Stager and Werker, 1997, experiments 2 and 3) in which children are familiarized with one novel word-object pairing (DEET). We modified the task to include two types of Switch trials, so that each participant was tested with two vowel contrasts (DIT and DOOT) rather than a single contrast relative to the familiarized word. Compared to Curtin et al. (2009), our version of the Switch task had a simpler familiarization phase, as they used two word-object pairings rather than one, and a more complex testing phase, with two Switch trials rather than a single Switch trial per participant. We chose a simpler familiarization phase in order to present two Switch trials during the test, which allowed us to compare the detection of a switch in two different vowels in the same infants. This was not possible in Curtin et al. (2009). We reasoned that this design will trigger word-object association performance, as Stager and Werker (1997, experiment 2) argued that 14-month-olds’ inability to notice the switch from BIH to DIH with this simplified procedure, despite their ability to perceptually discriminate the contrast /b/–/d/, was due to their treatment of the procedure as a word-object association task.

**FIGURE 1:**
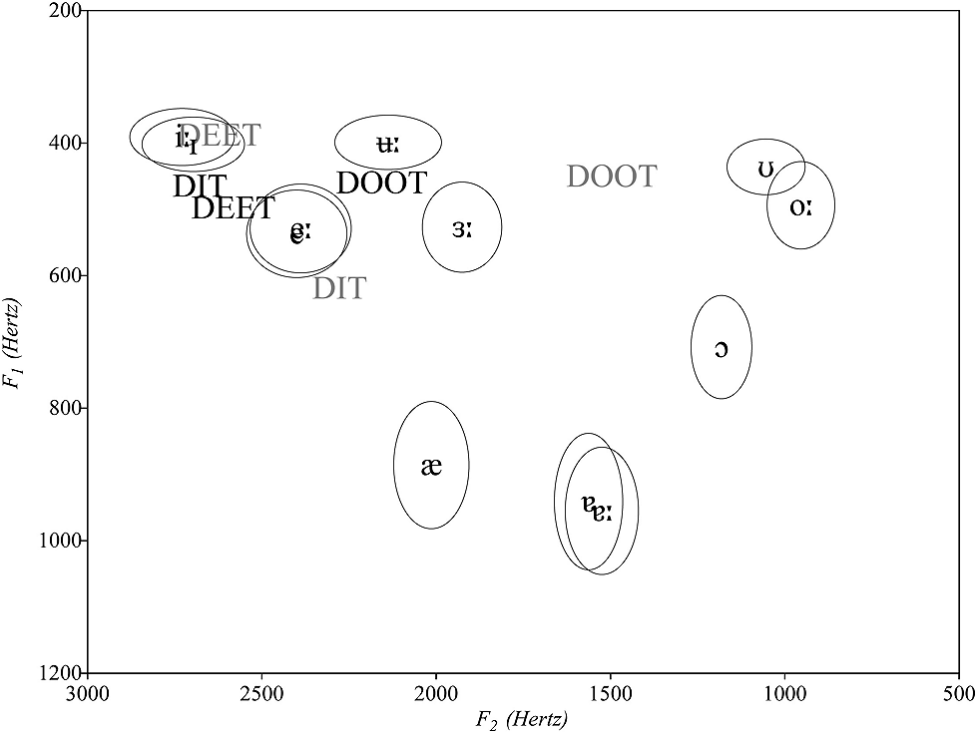
**Familiarization image **(A)** and pre- and post-test image **(B)**.** Visual stimuli were the same as those used in Curtin et al. (2009).

Our interest in examining accent differences stems in part from recent findings that the accent of both speaker and listener markedly shapes native and non-native vowel perception in adults (Escudero and Boersma, 2004; Escudero and Chládková, 2010; Chládková and Podlipský, 2011; Chládková and Escudero, 2012; Escudero and Williams, 2012; Escudero et al., 2012), and recognition of words with accent-differing vowels in 15-month-olds (Best et al., 2009; Mulak et al., 2013). If these findings extend to 15-month-olds’ learning of novel vowel minimal pair words, it is expected that AusE children will behave differently than the CE children in Curtin et al. (2009). That is, since AusE and CE vowels have different phonetic realizations in F1/F2 space (Cox and Palethorpe, 2007, see Figure 1, below), AusE 15-month-olds trained on novel word-object pairings produced in the CE accent are likely to exhibit different patterns of early word learning than those shown by their CE-learning counterparts in Curtin et al. (2009). But will they show different levels of success across their native AusE vs. the unfamiliar CE accents?

Models of perceptual attunement to native categories such as Kuhl’s Native Language Magnet model (NLM; Kuhl, 1991, 1994) and Best’s Perceptual Assimilation Model (PAM; Best, 1994, 1995) predict ease in discrimination for native vowel contrasts, as infants become highly attuned to the specific properties of their native vowels by 6 months (Werker and Tees, 1984; Kuhl et al., 1992; Polka and Werker, 1994). While both models are well supported by perceptual data in children younger than 15 months, they do not specifically address word learning involving minimal pairs at this age (cf. Tsao et al., 2004; Kuhl et al., 2005). However, if their thesis that native language attunement streamlines perception is correct, it would seem likely that with regard to the present study, children’s performance on the word learning task would be optimal in the native accent condition, where vowels would map precisely onto native categories based on familiar information that children hear on a regular basis.

Other studies also support better performance on early word recognition across accents for native/familiar accents (for a review, see Cristia et al., 2012). For instance, 20-month-olds looked longer to the picture of the target word CAR when it was produced with a final rhotic (/ka/), which is the most frequent production in the children’s Bristol UK environment, than when it was produced without the rhotic (/ka/), a pronunciation that is less frequent in Bristol (Floccia et al., 2012). Similarly, Mulak et al. (2013) found that when 15-month-olds heard a familiar word produced in their native AusE, they looked at the target image longer than the distracter image, but looked at both images equally when the word was produced in an unfamiliar accent (Jamaican Mesolect English). However, exposure to unfamiliar pronunciations or accents may overrule this native accent advantage for recognition of both familiar and novel words. For instance, White and Aslin (2011) showed that 19-month-olds who were familiarized to word-object pairings in which the word was consistently produced with a different vowel (e.g., BLACK or BATTLE instead of BLOCK or BOTTLE), subsequently generalized this vowel change to other familiar word-object pairings (e.g., they looked longer at the picture of a SOCK than at a distractor picture when hearing the word SACK). Additionally, 24-month-olds were able to recognize novel words across native and unfamiliar non-native accents when word training was in the unfamiliar accent (Schmale et al., 2011), and recognized a novel word produced in their native and in an unfamiliar non-native accent after a 2-min exposure to stories produced in the unfamiliar accent (Schmale et al., 2012).

The purpose of our study is to examine word learning of minimally different novel words (e.g., DEET–DIT) produced in different accents, rather than the recognition of familiar words produced in novel accents (e.g., Best et al., 2009; White and Aslin, 2011; Floccia et al., 2012; Mulak et al., 2013). Since we present each infant with a single accent, our study is also different from Schmale et al. (2011, 2012), where novel word recognition was tested between accents (familiarizing infants with one accent and testing them with another). Instead, we aim to demonstrate that the specific acoustic-phonetic realizations of a particular accent determine early word learning success in the absence of word knowledge or accent familiarity. To that end, we compare the performance of two infant groups, each presented with a different accent.

We propose that infants’ ability to learn our novel word stimuli (produced in a single accent throughout familiarization and testing) will be explained by the magnitude of the phonetic distinction of minimally different words in the accent with which they are presented (CE or AusE), rather than by accent familiarity (AusE = familiar/native, CE = non-native/unfamiliar). Inspection of the specific phonetic properties of the vowels in DEET (/i/), DIT (//) and DOOT (/u/) produced by CE and AusE speakers leads us to predict that in a word-object associative task with high demands such as the Switch task, the former accent will lead to higher success than the latter in early word learners. This prediction is supported by the values shown in Figure 1 where it can be observed that while /i/ and // are largely distinguished by F1 differences in CE, the same vowels produced in AusE have very similar F1 and F2 values^1^. If infants rely only on F1 and F2 for distinguishing these two vowels, as suggested by Curtin et al. (2009), AusE children would be expected to better distinguish /i/ and // in the unfamiliar CE accent than their native accent. Similarly, the magnitude of the phonetic distinction along the F1 and F2 dimensions for /i/–/u/ appears larger for CE than AusE vowels, since /u/ is more fronted in AusE than in CE and is therefore even closer to /i/. In fact, AusE /u/^2^ can be produced as far front as /æ/ (though it is, of course, higher than /æ/), which means that the only back vowel characteristic that it retains is its rounding feature (Cox, 2006). If the phonetic magnitude hypothesis predicts early word learning, AusE children presented with novel words containing CE vowels will notice a difference between a switch in the vowel of the familiarized word DEET better than those presented with the novel words containing AusE vowels.

This prediction of higher success for AusE children on CE novel words compared to AusE novel words that differ in the vowels /i/, // and /u/ is in line with the LP and PAM models which posit that listeners of any age classify vowel tokens based on their acoustic or articulatory properties, respectively. As shown in Figure 1, both CE // and /u/ have F1 and F2 values that are acoustically closer to other AusE vowels than to their phonemic counterparts. Specifically, CE // is a better acoustic match to AusE /𝜀/, while CE /u/ matches AusE //. Considered in terms of their articulatory properties, which mirror those of the acoustic patterns just described, the same pattern of assimilation is predicted by PAM. For an AusE listener then, the CE vowel contrasts /i/–// and /i/–/u/ should be perceived as the AusE contrasts /i/–/𝜀/ and /i/–//, which both display larger phonetic distinctions than the AusE phonemic counterparts /i/–// and /i/–/u/. Thus, AusE listeners should distinguish these two vowel contrasts. Given that the LP model proposes continuity between vowel perception at the end of the first year and word recognition early in the second year, AusE infants are likewise predicted to detect a switch from DEET to DIT and from DEET to DOOT in the unfamiliar CE accent. Such a finding would be in contradiction to the expectation and finding of the asymmetry in discrimination of these CE contrasts by CE children reported in Curtin et al. (2009), in which children detected a switch from DEET to DIT, but not DEET to DOOT (or DIT to DOOT).

## MATERIALS AND METHODS

### PARTICIPANTS

Participants were forty-eight 15-month-olds, who were randomly assigned to two groups: Twenty-four were familiarized and tested on CE stimuli (mean age = 15.26 months, range = 14.79–16.00 months; 12 girls) and 24 on AusE stimuli (mean age = 15.30 months, range = 14.79–16.10 months; 12 girls). All parents provided informed consent in accordance with the University of Western Sydney Human Research Ethics Committee. The infants were primarily Caucasian and from middle- to upper-middle-class AusE-speaking households in Sydney, Australia. Their amount of exposure to non-native languages or non-AusE accents ranged from 0 to no more than 12 h per week, none of which included the CE accent, as indicated by parental report. They were recruited via advertisements at pregnancy and parenthood fairs and parents’ magazines. Another 30 infants were tested but excluded from the final sample because of fussiness (*n*_AusE_ = 16; *n*_CE_ = 3), parental interference (*n*_CE_ = 1), pre-existing hearing loss (*n*_AusE_ = 1), obstruction of gaze from experimenter (*n*_AusE_ = 1) or because they did not meet the habituation criterion (*n*_AusE_ = 6; *n*_CE_ = 2).

### STIMULI AND APPARATUS

Participants were exposed to three CVC non-words during the task, namely DEET (/dit/), DIT (/dt/) and DOOT (/dut/). The CE stimuli were the same as those used in Curtin et al. (2009), which were produced by a female native speaker of CE. For the present study, we recorded a female native speaker of AusE who produced the same three CVC non-words. Both sets of stimuli were recorded at a 44 kHz sample rate directly onto a computer.

It was discovered that in the set of tokens for DEET, DIT, and DOOT used in Curtin et al. (2009), the first three and last three tokens were identical. This was mirrored when developing the AusE stimuli for the current study, such that both the CE and AusE speakers produced seven tokens of each CVC item, using the same range of infant-directed contours, with the first three tokens repeated at the end to create 10 tokens. The AusE speaker used the CE stimuli as models to match the F0 (fundamental frequency) contours as closely as possible. Following Curtin et al. (2009), infants were presented with a single sound file for each of the three words. The AusE sound files mirrored the CE sound files in token sequence (i.e., sequence of intonation contours), inter-stimulus interval and total duration of 20 s.

While the difference in the production of the consonants surrounding the vowels (/d/ and /t/) across the two accents was negligible, the vowels were judged by the first three authors (two trained phoneticians, one a non-native speaker of English, the other a native speaker of northern-cities American English, and the third a native speaker of AusE) to differ perceptibly and substantially between the two accents. These observations were confirmed by the F1, F2, and F3 values of the vowels in the two accents shown in Figure 1 and Table 1^3^. The table also includes measures of vowel duration and F0. Formant measurements were taken from the midpoint of the vowel (50% of total vowel duration).

**Table 1:** Average formant values, F0, and vowel duration for the vowels in the native accent (AusE) and unfamiliar accent (CE).

	Australian English (AusE)			Canadian English (CE)		
	DEET /i/	DIT //	DOOT /u/	DEET /i/	DIT //	DOOT /u/
F1	498.7 (72.4)	465.5 (60.6)	461.0 (80.6)	389.1 (44.8)	620.2 (73.4)	451.4 (42.3)
F2	2581.9 (226.9)	2677.5 (66.1)	2156.2 (178.3)	2622.2 (121.5)	2276.8 (111.1)	1496.2 (114.7)
F3	3193.6 (303.9)	3182.0 (246.8)	2719.7 (258.2)	3025.5 (182.5)	2937.8 (158.7)	2471.8 (199.6)
F0	273.8 (88.8)	311.8 (78.7)	265.9 (76.8)	312.9 (106.1)	271.5 (55.1)	272.4 (76.5)
duration	253.5 (51.7)	244.0 (59.9)	298.9 (99.2)	302.6 (42.1)	245.7 (28.7)	300.8 (38.5)

*Formant measurements (in Hz) were taken from the midpoint of the vowel (50\% of total vowel length). Duration is in ms. Values in parentheses represent one standard deviation from the mean.*

The values in Table 1 show that the CE stimuli indeed have larger intervocalic differentiation in F1 and F2 than the AusE stimuli, confirming our hypothesis that the acoustic features (or articulatory correlates) of CE vowels could be used as clearer cues to vowel discrimination than those of AusE vowels. Specifically, as shown in Figure 1 and discussed in the introduction, the vowels in the CE stimuli show larger phonetic distinctions than the vowels in the AusE stimuli, as the former stimuli have acoustic properties that match (“→”) those of highly distinct AusE vowels: CE DEET → AusE /i/ or //, CE DIT → AusE /e/, and CE DOOT → AusE //. Thus the prediction set forth by LP and PAM that CE vowels would be better discriminated than AusE vowels apply to the specific stimuli used in the present study.

**FIGURE 2:**
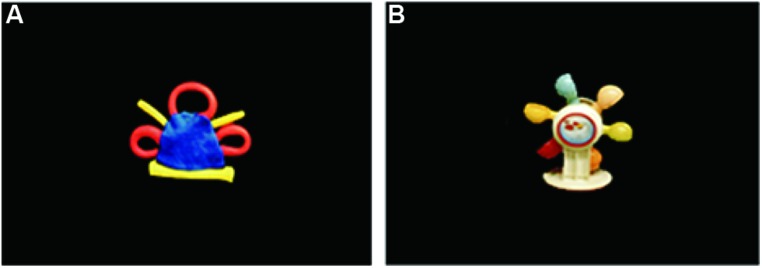
**Looking time to the Same (DEET) test trial, and two Switch trials (DIT, DOOT) for the AusE and CE stimuli groups.** Error bars represent one standard error.

The visual stimuli used in the familiarization and test phases were two of the images used by Curtin et al. (2009). One attractive novel object (see Figure 2A) was used for the familiarization phase (habituation) and test trials, and a toy waterwheel (Figure 2B) was used for both the pre- and post-tests. Similar to the presentation procedure in Curtin et al. (2009), the novel object moved back and forth across the screen at a slow and constant speed, while the waterwheel was filmed with its arms moving in a rotating motion.

### PROCEDURE

We used the simple version of the Switch design (Stager and Werker, 1997, experiments 2 and 3), which we modified to include two types of Switch trials rather than one so that each participant was presented with all three vowel contrasts. During familiarization to the novel word-object association, infants were presented with a single word-object pairing, which consisted of the crown object (Figure 2A) paired and ten tokens of the word DEET. As in Curtin et al. (2009), each familiarization trial had a duration of 20 s, where the infants heard a sound file containing 10 tokens of the word DEET produced by either the CE speaker or the AusE speaker. Each trial started when the infant looked at a looming attention getter. Looking time to the screen for each trial was coded online, and familiarization trials repeated until participants reached a pre-set fixed habituation criterion (two consecutive trials with <65% of looking time from the average of the first two trials). Once the habituation criterion was met, three test trials were presented, each of them starting when the infant looked at a looming attention getter, as during familiarization. In the Same trial, the same 10 tokens of the word DEET and the crown object were presented. In the two types of Switch trials, the pairing was violated. That is, infants saw the same object moving but heard ten tokens of a different word in each Switch trial: DIT or DOOT.

As in previous early word learning studies that used the Switch design, if infants do not recognize the auditory word presented in a Switch trial to be different from the word presented to them during familiarization, the Same (DEET) and Switch trials (DIT or DOOT) would be equally familiar, resulting in equal looking times for both types of trials. This would be interpreted as infants’ failure to discriminate the vowel in familiarization trials (DEET) from the vowel in the Switch trial (DIT or DOOT). Conversely, if infants do recognize that the auditory word presented in the Switch trial is different than the word presented in the familiarization trials, they would look longer to Switch than Same trials, which would be interpreted as discrimination of the vowels presented in the Switch trials. In order to rule out the possible effect of order of Same and Switch trials, infants in both the CE and AusE stimulus condition were presented with three different orders for the test trials: (1) DEET–DOOT–DIT (Same–Switch1–Switch2), (2) DOOT–DEET–DIT (Switch1–Same–Switch2), and (3) DIT–DOOT–DEET (Switch2–Switch1–Same). Each accent × order group contained four infants (two females, two males).

The familiarization and test trials were preceded (pre-test trial) and followed (post-test trial) by a trial in which the waterwheel object (Figure 2B) was presented together with 10 tokens of the novel word LARD^4^, produced by a different female AusE speaker in infant-directed speech. This was to ensure that the infants recovered (i.e., showed an increase in looking time) when presented with a large acoustic-phonetic change in the auditory word and visual referent, indicating that they were not fatigued or generally disinterested in the task.

## RESULTS

We first analyzed levels of attention during the pre- and post-test trials as well as performance during familiarization to assure that group differences during testing were not attributable to differences in their overall attention or in their rate of habituation. With respect to overall attention to the task, a mixed 2 (trial: post-test vs. last familiarization trial) × 2 (stimulus: CE vs. AusE) analysis of variance (ANOVA) revealed a significant effect of trial [*F*(1,46) = 371.11, *p* < 0.001; $\eta_{p}^{2}$ = 0.89], with infants looking longer to the post-test trial (*M* = 18.31 s, *SD* = 1.71) than to the average of the last two familiarization trials (*M* = 7.89 s, *SD* = 3.18), and there was no interaction with accent. Thus, infants’ engagement in the task persisted until the end of the experiment in both accent conditions. Regarding their performance during familiarization, an independent-samples *t*-test revealed no difference in average looking time to the last two familiarization trials across accent conditions [*t*(46) = -0.92, *p* = 0.363, 95% CI (-2.12, 0.79)]. Furthermore, an independent-samples *t*-test on the number of familiarization trials, which were between 4 and 24 for all infants (*M* = 8.88, *SD* = 4.33), did not differ between CE and AusE stimulus conditions [*t*(46) = 0.20, *p* = 0.84, (-2.29, 2.79)]. Together, these results suggest that neither overall looking time nor degree of habituation were different across the accent groups and are therefore not predictive of differences during testing.

To test our prediction that detection of a switch in the test trials would differ between the two accent groups, we conducted a repeated measures ANOVA using looking time during test trials as the dependent variable, with test trial (Same = DEET vs. Switch = DIT vs. Switch = DOOT) as a within-subject factor, and accent of the stimuli (CE vs. AusE) and order of test trials (DEET–DOOT–DIT vs. DOOT–DEET–DIT vs. DIT–DOOT–DEET) as between-subjects factors. This revealed a main effect of test trial [*F*(2,84) = 4.55, *p* = 0.013, $\eta_{p}^{2}$ = 0.10], as well as a trend toward a main effect of order of test trials [*F*(2,42) = 2.98, *p* = 0.062, $\eta_{p}^{2}$ = 0.12]. Participants who received the test trials in the order DOOT–DEET–DIT looked longer during the test overall compared to participants who received trials in the order DEET–DOOT–DIT [*t*(31) = 2.08, *p* = 0.043, 95% CI (0.08, 5.35)] or DIT–DOOT–DEET [*t*(31) = 2.15, *p* = 0.038, (0.17, 5.43)]. There was also a trend toward an interaction between test trials × accent [*F*(2,84) = 2.82, *p* = 0.065, $\eta_{p}^{2}$ = 0.06]. Independent-samples *t*-tests comparing looking time to each test trial between accent conditions revealed no significant difference in looking time to test trials between accents, but a trend toward longer looking to DEET (Same) in AusE relative to the CE condition [*t*(46) = -1.83, *p* = 0.074, (-4.70, 0.22)].

To follow up the main effect of test trial, we conducted simple effects tests comparing participants’ looking time to each of the Switch trials (DIT, DOOT) with looking time to the Same trial (DEET). Looking time was greater for DIT (Switch; *M* = 10.56 s, *SD* = 4.60) than for DEET [Same; *M* = 9.32 s, *SD* = 4.34; *F*(1,42) = 4.84, *p* = 0.033, $\eta_{p}^{2}$ = 0.10], and was greater for DOOT (Switch; *M* = 10.45 s, *SD* = 4.71) than for DEET [Same; *F*(1,42) = 8.62, *p* = 0.005, $\eta_{p}^{2}$ = 0.17].

**FIGURE 3:**
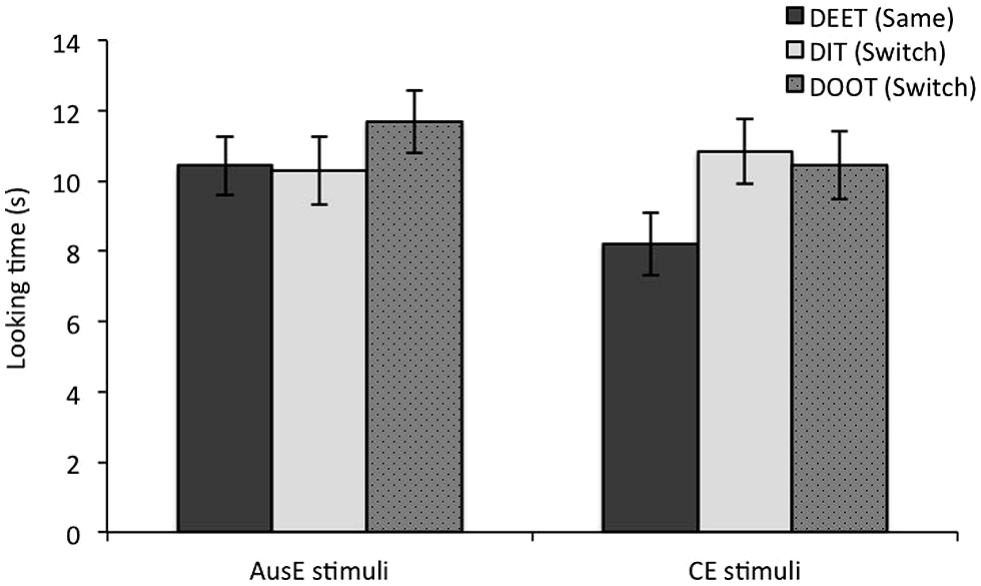
**Average spectral change for the vowel /i/ in the ten familiarization tokens of DEET for the two accents.** The accent label and the end of each line are plotted at the average formant frequency (across tokens) at 75% of the vowel duration, and each line originates at the average formant frequency at 25% of the vowel duration. There was a larger movement of the formants across the 25 and 75% points of the vowels in the AusE than in CE.

For our specific prediction that participants would show a greater magnitude of difference in looking time to Switch trials relative to the Same trial for the CE than for the AusE stimuli condition, we carried out simple effect tests on participants’ performance on each test trial for the CE and AusE conditions separately. As can be seen in Figure 3, participants in the CE condition had longer looking times for DIT (Switch; *M* = 10.84 s, *SD* = 4.49) than for DEET [Same; *M* = 8. 20 s, *SD* = 4.34; *F*(1,23) = 8.66, *p* = 0.007, $\eta_{p}^{2}$ = 0.27], and for DOOT (Switch; *M* = 10.45 s, *SD* = 4.71) than for DEET [Same; *F*(1,23) = 6.39, *p* = 0.019, $\eta_{p}^{2}$ = 0.22]. In contrast, for participants in the AusE condition, simple effects tests showed that there was no difference between looking times to DIT (*M* = 10.28 s, *SD* = 4.75) and DEET [*M* = 10.44 s, *SD* = 4.13; *F*(1,23) = 0.49, *p* = 0.827, $\eta_{p}^{2}$ < 0.01], or between DOOT (*M* = 11.69 s, *SD* = 4.38) and DEET [*F*(1,23) = 2.28, *p* = 0.145, $\eta_{p}^{2}$ = 0.09]. Thus, participants in the CE condition distinguished both DIT and DOOT from DEET, while those in the AusE condition did not make either of these two distinctions.

**FIGURE 4:**
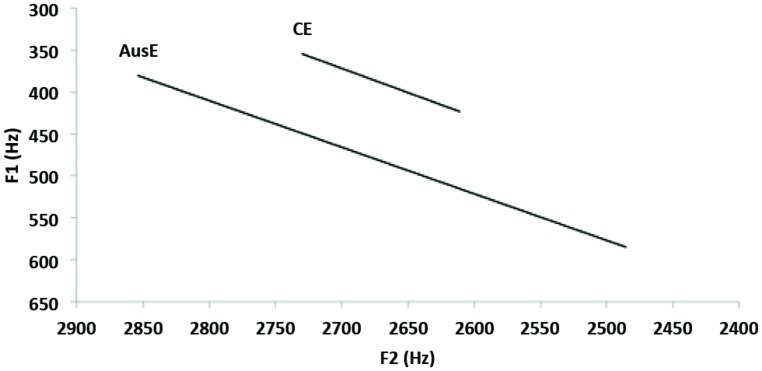
**Average spectral change for the vowel /i/ in the ten familiarization tokens of DEET for the two accents.** The accent label and the end of each line are plotted at the average formant frequency (across tokens) at 75% of the vowel duration, and each line originates at the average formant frequency at 25% of the vowel duration. There was a larger movement of the formants across the 25 and 75% points of the vowels in the AusE than in CE.

To determine whether there were differences in spectral variation across the CE and AusE word DEET, which may have been responsible for the differential performance in the two accent conditions, measures of F1 and F2 were taken at 25 and 75% of the vowel for each of the 10 familiarization tokens. Using F1 and F2 measures as the dependent variables, we ran two (2) × 2 ANOVAs, with time (25, 75%) as a within-subjects factor, and accent (AusE, CE) as a between-subjects factor. For the F1 measure, there was a main effect of time [*F*(1,18) = 38.16, *p* < 0.001, $\eta_{p}^{2}$ = 0.68] and accent [*F*(1,18) = 15.19, *p* = 0.001, $\eta_{p}^{2}$ = 0.68], as well as a time × accent interaction [*F*(1,18) = 9.43, *p* = 0.007, $\eta_{p}^{2}$ = 0.34]. For the F2 measures, there was a main effect of time [*F*(1,18) = 83.39, *p* < 0.001, $\eta_{p}^{2}$ = 0.82] and a time × accent interaction [*F*(1,18) = 21.93, *p* < 0.001, $\eta_{p}^{2}$ = 0.55]. As can be seen in Figure 4, spectral change is much larger for the DEET vowel in the AusE than in the CE stimuli. This larger variation within the 10 AusE tokens may explain the longer looking times to DEET Same trials during the test phase, as participants may have treated some AusE tokens as containing different vowels. In that respect, it is worth mentioning that five of the seven infants who did not meet criterion were in the AusE condition, which indicates that a larger number of infants in this condition relative to the CE condition failed to habituate to their DEET trial.

## DISCUSSION

This study compared AusE-learning 15-month-olds’ ability to learn a novel word-object pairing (DEET) and subsequently distinguish it from pairings that included the same referent object, but switched the spoken word to two words that differed from the original word by their vowel (DOOT and DIT). The novel word and two foils were produced in either the participants’ native AusE accent, or an unfamiliar accent, CE. The young word learners distinguished the newly learned word from the two vowel-differing alternates when words were spoken in CE, but not when they were produced in their native AusE accent. That is, only children who heard the CE words showed a recovery in looking time from the Same trial to the Switch trials.

These results demonstrate for the first time that children younger than 17 months can distinguish minimal vowel pairs in which the vowels primarily differ along acoustic dimensions other than F1. Curtin et al. (2009) found that CE-learning 15-month-olds discriminated only the contrast /i/–//, which is primarily differentiated in F1. Based on this, the authors proposed that F1 has special status in vowel discrimination in early word learning, and speculated that this may be due either to F1 having more energy in the speech signal compared to F2 and F3, or to F1 differentiating a wide range of vowel contrasts in CE. Here, AusE-learning 15-month-olds noticed a change from the familiarized DEET stimulus regardless of whether the Switch-trial vowels differed mainly in the F1 dimension (DEET–DIT) or F2 dimension (DEET–DOOT). This contradicts the findings of Curtin et al. (2009) and their proposal that F1 is more important than F2 in vowel discrimination by children of this age. It seems that the utilization of phonetic detail in early word learning is not universal, but rather is dependent on how phonetic dimensions are perceived by specific listener groups based on their native accent experience.

Alternatively, the different findings across studies could be explained by their different procedures. Specifically, despite the fact that Stager and Werker (1997) also found word learning difficult with the single word-object version of the Switch task used in the present study, this simpler familiarization phase may have triggered word discrimination rather than word-object association in our study. This possibility is unlikely, however, as it would suggest that two groups of infants of the same age used different processing strategies when presented with the same task, i.e., discrimination for the group presented with CE stimuli and word-object association for the group presented with AusE stimuli. Future studies should further explore this possibility by presenting CE infants with our single-word familiarization or AusE infants with Curtin et al.’s (2009) two-word familiarization. Further research should also examine the possibility that infants might resort to different processing strategies for stimuli produced in their native vs. a non-native accent.

The present findings showing that 15-month-olds detect differences in vowel minimal pairs is in contrast with work showing that children under 17 months are unable to learn novel vowel minimal pairs in an interactive object reaching task (but do learn novel consonant minimal pairs; Nazzi, 2005; Nazzi and New, 2007; Havy and Nazzi, 2009; Nazzi et al., 2009). As discussed in Curtin et al. (2009), this disparity may be due to differences between Nazzi and colleagues’ interactive object-reaching task, which used live pronunciations in a natural sentential context by speakers interacting with the participants, and the task used in the present study, which used previously recorded strings of single word utterances. It may be that when interacting with a real speaker, children younger than 17 months relax their tolerance for vowel variation in a way that they do not for consonants or for less interactive settings. Additionally, as the stimuli in the present study were comprised of strings of single words differing only in their vowel, this may have focused children’s attention to the vowel differences in a way that would be less likely to occur in a more natural language setting.

The most striking finding is that AusE children’s success with F1 and F2 minimal pair vowel distinctions was limited to words produced in the unfamiliar CE accent. The NLM model (Kuhl, 1991, 1994) would predict that familiarity with words and vowels in the native accent should lead to better discrimination of minimal pairs in the native accent compared to an unfamiliar accent. Our findings also pose a substantial challenge to exemplar models and other models of early word learning that rely on tracking of statistical distributions in the input (e.g., Saffran et al., 1996). Such models cannot explain why young children fail to distinguish minimal pairs in the Switch task when the words are produced in their native accent, but succeed when they are produced in an unfamiliar accent. This is in part because neither approach explicitly considers how the cognitive demands of the experimental task may affect performance, specifically that some tasks may make it more difficult to pay attention to small phonetic differences in early word learning.

Our results support the phonetic magnitude hypothesis that we put forward in the introduction, which posits that in a demanding task, such as word learning for novice learners, the magnitude of the phonetic distinction between two vowel sounds predicts successful learning and discrimination of vowels in a word learning context (Curtin et al., 2009). This appears to occur irrespective of the regional accent spoken in the native environment. As can be seen in Table 1, the F1 and F2 distinctions between the vowel contrasts are greater in CE than in AusE. The AusE-learning infants distinguished the CE vowel minimal pairs, but their performance was less reliable when listening to the same vowel contrasts in their native AusE accent. Our study thus shows that if an infant is presented with novel word-object pairings in only one accent, rather than novel words across accents (Schmale et al., 2011, 2012), minimally different words that are distinguished by a large phonetic contrast are easier to learn than those with a smaller phonetic contrast, regardless of whether the accent in which the words are produced is familiar or novel.

Specifically, we believe that the small phonetic difference between AusE vowels, rather than a difference in performance by infants across accent groups, better explains our results given the much larger attrition rate for infants in the AusE vs. CE condition. As shown in the participants section, 22 infants in the AusE condition were excluded from analysis because of either fussiness during the experiment or because they did not meet the habituation criteria, while only 5 infants in the CE condition were excluded for the same two reasons. Thus, infants had more trouble performing the task when presented with the AusE than with the CE stimuli, suggesting difficulty processing the native AusE stimuli.

Furthermore, recent results from our lab (Escudero et al., accepted) demonstrate that AusE adult listeners also have difficulty learning the same AusE vowel minimal pairs of the present study. Adult AusE listeners were tested on their ability to identify the correct word-object associations after a short exposure to word-object referent pairs that could only be inferred across trials. They had fewer correct answers to minimal pairs involving the words DIT, DEET, DOOT, and DUT than when the minimal pairs involved the words BON, DON, PON, and TON (used to identify consonant minimal pairs). Since the vowel minimal pairs included the same vowels and consonants presented in the current study, it can be concluded that these AusE vowel minimal pairs are difficult to perceive even for native-accent adult listeners. Although the vowels in the CE stimuli do not have properties that are exactly the same as the acoustically closest AuE vowels (Figure 1), and would therefore be less frequent in the AusE infants’ linguistic environment, the magnitude of their phonetic contrast is much larger than that of their AusE counterparts, and according to our phonetic magnitude hypothesis and our results, easier to discriminate and use in early word learning. It remains to be tested whether AusE adults also have less trouble learning the same vowel minimal pairs when produced in another regional accent of English in which the magnitude of the same vowel contrasts is larger (e.g., CE or American English). That would mean that our phonetic magnitude hypothesis might apply across the lifespan when task demands are high, for instance, when having to demonstrate word learning after only a few minutes of exposure in an implicit learning task.

The findings are in line with the LP model, which can be considered a theoretical and computational implementation of the phonetic magnitude hypothesis (Boersma et al., 2003; Escudero and Boersma, 2004; Escudero, 2005, 2009). The LP model asserts that infants’ vowel categories are emergent and based on the specific auditory dimensions that are most salient to infants depending on their native accent and their age. This means that adults, children, and infants exposed to different accents are likely to differ in the way they weight the auditory dimensions of any given vowel token (native or non-native). Within the model, the saliency or perceptual weight of a phonetic dimension, such as F1 or F2, depends on the magnitude of the phonetic difference it offers in a specific accent. It is proposed that young infants, who do not yet have a well-developed lexicon, may concentrate on the most salient phonetic cue, while ignoring other less salient ones. From an LP perspective, AusE children are exposed to very small differences in F1 and F2 in the production of their native vowels /i/, // and /u/, and therefore hear large enough differences between the CE productions of the same vowels along both dimensions, which explains why they more easily discriminate them. In contrast, CE infants are exposed to larger F1 than F2 distinctions for these three vowels, which is the explanation given in Curtin et al. (2009) for their asymmetric findings. Thus, the reason why AusE children rely on both F1 and F2 for the CE stimuli is because both dimensions are as salient to them, while the same two dimensions are equally difficult to distinguish in the AusE stimuli. Following the LP model, we predict that CE infants would have the same failure to distinguish AusE vowels as AusE infants, due to the small, non-salient contrast for F1 and F2 in the AusE vowels.

The PAM model presumes that native categories are in place by 15 months, but that they have not yet necessarily become phonological contrasts used for differentiation of words. Instead, these more advanced lexical skills appear to emerge later on, and are associated with the expressive vocabulary expansion that occurs around 19 months (Best et al., 2009; Mulak and Best, 2013; Mulak et al., 2013). At 15 months, discrimination of native and non-native segments is dependent on mappings to L1 categories. While this could predict better performance in the native accent, it may be that the AusE-learning children perceived the CE /i/–// vowel contrast as corresponding to the phonetically larger AusE /i/–/𝜀/ contrast, and the CE /i/–/u/ contrast to the phonetically larger AusE /i/–// contrast (see Figure 1).

Under high cognitive load, such as in the word learning task of the present study, reliable phonetic cues may play a larger role, in line with both LP and PAM. The results of this study are thus consistent with performance being linked to unidimensional distinctions between vowels, as proposed within the LP framework, rather than the multidimensional approach in adult listening. This holds regardless of whether each stimulus dimension is characterized in terms of acoustic dimensions (F1 and F2 values: LP) or articulatory distinctions (vowel height and jaw opening: PAM). Further research should show whether the use of reliable phonetic cues is a developmental stage in L1 phonological acquisition, as proposed by the LP model, a strategy used in highly demanding word-learning situations, or a combination of both.

In sum, these results show that success in early word learning depends on the magnitude of the phonetic (acoustic or articulatory) distance between novel vowel minimal pairs, and not on familiarity with the specific productions of the words (native vs. non-native accent), nor on the universal salience of a specific acoustic dimension (e.g., F1 vs. F2). Current models of early language development should consider the role of phonetic distance in perceptual and lexical development and how this may vary as a function of task demands.

## Conflict of Interest Statement

The authors declare that the research was conducted in the absence of any commercial or financial relationships that could be construed as a potential conflict of interest.

## Footnotes

1 In adult speech, the AusE vowels seem to be distinguished instead mostly by subtle diphthongization (/i/ can be produced with a small “onglide” or delayed target which gives it the quality of a diphthong) and duration (Cox and Palethorpe, 2007; see Figure 2 in Cox, 2006). Curtin et al. (2009) also showed that in their CE stimuli, which were produced in child-directed speech, /i/ and /ı/ had overlapping duration values since in this speech style all CE vowels are apparently lengthened to similar extents. The authors show that duration is therefore an unreliable cue for this contrast in CE. Duration differences among these vowels are likely to also be unreliable in AusE child-directed speech, as is evident in **Table 1**.

2 To more accurately reflect its phonetic characteristics, centralized and rounded [u-] is commonly used to represent AusE /u/ (Harrington et al., 1997; Cox, 2006).

3 Although the first three and last three tokens are identical in the set of 10 tokens for DEET, DIT, and DOOT in both AusE and CE, formant averages are based on all 10 tokens so that the averages reflect all the tokens that infants heard during familiarization.

4 As LARD occurs at a low frequency in adult vocabularies, it is not expected to be part of the 15-month-old lexicon and is thus regarded here as a novel word.
